# Phenytoin-induced dyskinesia: a case report

**DOI:** 10.1186/s13256-023-04033-6

**Published:** 2023-07-21

**Authors:** Kashvi C. Shah, Nishi S. Patel, Paritosh Vasani, Avinash Khadela, Vivek P. Chavda, Lalitkumar Vora

**Affiliations:** 1 L. M. College of Pharmacy, Ahmedabad, Gujarat 380009 India; 2 GCS Medical College, Hospital and Research Centre, Ahmedabad, Gujarat 380025 India; 3 Department of Pharmacology, L. M. College of Pharmacy, Ahmedabad, Gujarat 380009 India; 4 Department of Pharmaceutics and Pharmaceutical Technology, L. M. College of Pharmacy, Ahmedabad, Gujarat 380008 India; 5 School of Pharmacy, Queen’s University Belfast, 97 Lisburn Road, Belfast, BT9 7BL UK

**Keywords:** Phenytoin, Dyskinesia, Seizures, Adverse drug reaction

## Abstract

**Background:**

Dyskinesia is a movement disorder categorized by involuntary movement of muscle. Although dyskinesia can be brought on by taking medications, it can also be a symptom of a variety of diseases. Antiepileptic drug-induced involuntary movements have been well researched. Rare reports have been made for dyskinesia, a type of dystonia caused by phenytoin. The mechanism of its occurrence must be succinctly studied.

**Case presentation:**

A 53-year-old Asian patient taking phenytoin (100 mg twice daily) experienced symptoms of perioral muscle involuntary movement, impaired speech, and generalized tremors and was admitted to the hospital. Brain magnetic resonance imaging showed significant development of encephalomalacia and porencephaly. The serum phenytoin levels were in the toxic range (33 g/ml). These were suggestive of phenytoin-induced dyskinesia. Levetiracetam and clonazepam were initiated, and the patient showed significant improvement in the symptoms.

**Conclusion:**

This case presented a substantial reference value for the differential diagnosis and treatment prognosis of phenytoin-induced dyskinesia. The phenytoin-induced dyskinesia in this patient was successfully reversed with prompt identification and treatment. According to the case study’s findings, such people may benefit from periodic therapeutic drug monitoring.

## Introduction

Phenytoin (also known as 5,5-diphenylhydantoin) is an aromatic hydantoin derivative used to treat prophylactic seizures and posttraumatic seizures. It aids in the regulation of seizures by blocking voltage-gated sodium channels and inhibiting neuronal firing [1]. Despite having complicated and unpredictably varying pharmacokinetic features, phenytoin is often safe, effective, affordable, and relatively easy to use. Ataxia, diplopia, nystagmus, vertigo, mental confusion, hallucinations, blurred vision, mydriasis, cerebral atrophy, cerebral malfunction, migraines, and sleeplessness are common adverse effects involving the central nervous system [2]. Phenytoin-induced dyskinesia is uncommon, although it is prevalent with other antiepileptic drugs. Various involuntary movement abnormalities, such as orofacial and limb dyskinesia, shaking, asterixis, hemiballismus, dystonia, and myoclonias, can be brought on by phenytoin intoxication [3].

## Case report

**Fig. 1:**
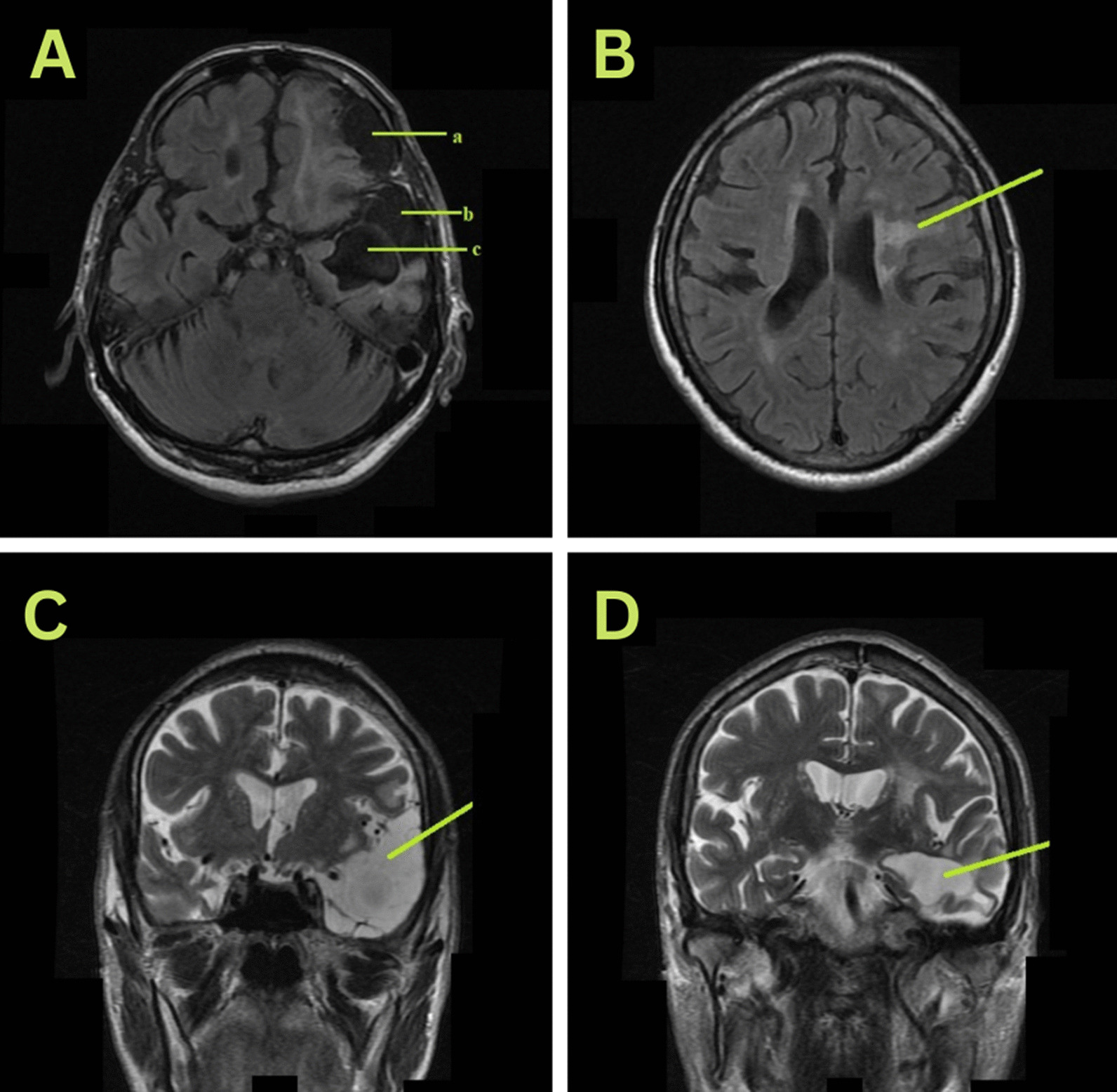
**A** Axial T2 view: The yellow bar a. Shows encephalomalacia in the left frontal lobe, b. and c. Shows encephalomalacia in the left temporal lobe. **B** (Axial T2 view): The yellow bar reveals cerebral atrophic changes. **C** (T2 coronal view): The yellow bar indicates left temporal lobe encephalomalacia with the development of porencephaly. **D** (T2 coronal view): The yellow bar shows cerebral atrophic changes

A 53-year-old Asian right-handed male patient with a 20-year history of a road traffic accident (RTA) was started on 100 mg Eptoin (phenytoin) twice a day. The patient was admitted to a hospital with symptoms of perioral muscle involuntary movement, impaired speech, and generalized tremors. On examination, his vital signs and laboratory investigations were within the normal range. A brain magnetic resonance imaging (MRI) scan indicated that the patient had encephalomalacia with surrounding gliosis affecting the left temporal and bilateral basifrontal lobes with secondary development of porencephaly. It also revealed evidence of dilatation of the ventricular system suggestive of cerebral and cerebellar atrophic changes (Fig. 1). MR angiography of intracranial and extracranial vessels revealed no noticeable findings. On the third day, the serum phenytoin level was 33 g/ml (10–20 g/ml). Adverse drug reaction (ADR) causality evaluations using the World Health Organization (WHO) causality assessment scale and Naranjo’s Algorithm revealed a “probable” time–temporal link of ADR. On the first day, the ADR was managed by withdrawing phenytoin from the regimen. He was given supportive treatment in the form of levetiracetam 500 mg and clonazepam 0.5 mg twice daily and thrice daily, respectively. For three days, the patient was kept hydrated with intravenous fluids. The patient was routinely monitored. By the third day after discontinuing phenytoin, involuntary movements had stopped.

## Discussion and conclusion

Antiepileptic drugs such as carbamazepine [4], phenobarbital [5], felbamate [6], lamotrigine, ethosuximide, valproic acid [7], gabapentin, and phenytoin have all been linked to reported involuntary movements. Commonly reported involuntary movements include choreoathetosis, dystonia, dyskinesia, and ballismus [8]. Phenytoin-induced involuntary movements are extensively documented, with rare occurrences of dyskinesia being reported [9]. Phenytoin-induced dyskinesia appears to be focal or generalized and can persist for hours, days, or even years. Symptoms have been reported to disappear after cessation of phenytoin therapy [10]. This case report sheds light on evidence of phenytoin-induced dyskinesia. Our patient is a 53-year-old man who has been reportedly taking phenytoin for over 20 years and experienced dyskinesia while on the drug, which completely disappeared when the drug was withdrawn. His MRI scan revealed encephalomalacia with surrounding gliosis affecting the left temporal and bilateral basifrontal lobes.

Harrison *et al*. investigated the demographic distribution of patients suffering dyskinesia owing to phenytoin intoxication and found that 50% were less than 20 years old, while only 20% were older than 40 years old [11]. Similar findings were found in a study performed by Montenegro *et al*., which found that children have a 5–6 times greater risk of phenytoin-induced dyskinesia than adults [12].

Phenytoin is a narrow therapeutic index drug with an atherapeutic range from 0 to 20 μg/ml. Elimination of phenytoin follows mixed-order kinetics; it follows first-order kinetics until ≤ 10 μg/ml, and above 10 μg/ml, it follows zero-order kinetics. Thus, the elimination profile of phenytoin predisposes the patient to develop adverse drug reactions, and increased half-life due to zero-order pharmacokinetics results in prolonged duration of toxic symptoms [13].

The pathophysiology of phenytoin-induced dyskinesia is not well understood. Harrison *et al*. postulated that there is a disruption in the functional balance of the basal ganglia output systems, possibly because of phenytoin’s differential influence on dopaminergic activity [11]. The most commonly recognized theory is that underlying brain lesions or gliosis enhance dopaminergic and serotonergic activity in the striatum, and individuals with these abnormalities are more likely to have this negative impact [3].

Both the WHO’s causality assessment algorithm and Naranjo’s Scale were used to determine causation. Based on a time–temporal link and the reaction being unlikely to be attributable to any contemporaneous condition or medication, the WHO scale rated it “probable,” and Naranjo’s Scale rated it the same with a score of 7.

According to Hussein *et al*., there may be no correlation between blood levels of antiepileptic medications and toxicity. As a result, the serum level of phenytoin may be non-toxic [14]. It is critical to consider phenytoin as a differential diagnosis in any patient who exhibits involuntary movements while on phenytoin medication. This is a case with a great prognosis following drug discontinuation/withdrawal.

## Data Availability

Not applicable.
